# Sensitivity Analysis in Meta‐Analysis: A Tutorial

**DOI:** 10.1002/cesm.70067

**Published:** 2026-01-05

**Authors:** Nyan Min Aung, Ivan Jurak, Seemab Mehmood, Emma Axon

**Affiliations:** ^1^ Department of Oral Biological Science University of Dental Medicine Mandalay Myanmar; ^2^ Department of Physiotherapy University of Applied Health Sciences Zagreb Croatia; ^3^ Fatima Jinnah Medical University Lahore Pakistan; ^4^ Evidence Production and Methods Department Cochrane Methods Support Unit, Cochrane London UK

## Abstract

This tutorial explains when systematic review authors may consider performing a sensitivity analysis in a meta‐analysis. Such scenarios include removing studies at high risk of bias, exploring the effect of outliers and examining differences in study characteristics (e.g., participants’ age, study design). In addition, examples are provided, as well as advice on how to interpret and report the results. The tutorial also explains the differences between subgroup and sensitivity analyses, as well as describing the disadvantages of a sensitivity analysis. To support this tutorial, a link to an online module, which includes videos and quizzes, is also provided.

## 1 What Is Sensitivity Analysis?

Even when a systematic review follows a rigorous research methodology, its robustness is limited by characteristics and quality of included studies. When encountering such limitations, authors can consider several analytical decisions, such as combining or excluding studies, selecting statistical models, or handling missing data. In this context, a sensitivity analysis is used to evaluate how these decisions and underlying assumptions influence the overall conclusion of a systematic review [1]. In essence, a sensitivity analysis acts as a repeat of a meta‐analysis, by which the robustness of findings from the main meta‐analysis is evaluated. However, it do not guarantee the findings are free from influence of the factors outside of the authors’ control or from methodological weaknesses in the individual studies.

## 2 When Should They be Used?

Examples of assumptions include [1]:

I. **Variations in risk of bias of included studies**

− For example, excluding trials that have a high risk of bias.

II. **Variations in effect size or influence of outliers** which can be defined as the studies with an apparent difference in magnitude or direction compared with the other included studies

− For example, following the main meta‐analysis, the review authors may observe an obvious outlier, such as a trial that has an exaggerated effect of intervention. Then, they may decide to remove it to see its impact on the size of the overall estimate.

III. **Variations in PICO** (Populations, Interventions, Comparisons, and Outcomes)

− Variations in study PICO elements can be frequently investigated through subgroup analyses (further details in Table 2). However, sensitivity analyses can also be planned to test the robustness of the findings under different assumptions of PICO.
− For example, a meta‐analysis may include studies with different populations (such as children with and without special educational needs). A sensitivity analysis could be used to restrict the analysis to those studies that only include children without special educational needs.

IV. **Heterogeneity**

− Frequently, studies included in a meta‐analysis may differ in clinical context, methodological setting and statistics. Each of the variations can influence the pooled estimate. As a result, a sensitivity analysis can be used to investigate the cause of this heterogeneity.

V. **Exploring the impact of imputing or excluding missing data**

− The term missing data may refer to several situations: patient drop‐out, missing summary measures, missing specific design parameters such as the intra‐class correlation coefficient, missing individual participant data (IPD), missing of the entire study, and so on.
− Imputing data is a statistical method used to replace missing data with substituted values.
− For example, review authors may decide to impute missing data from a trial by assuming data is not missing at random (see Section 2 for further details). A sensitivity analysis could be conducted to explore how this decision impacts the size of the overall effect.
− As another example, the intra‐class correlation coefficient (ICC) may not be reported in cluster randomized trials. The reviewers can borrow this value from similar studies or define it in a reasonable range (see Cochrane Handbook [1] Chapter 10). Then, they can perform sensitivity analyses using different values for the ICC.

VI. **Variations in analytical methods**

− For example, exploring the impact of using different meta‐analysis models (Fixed or Random effects or other statistical models) on the overall estimate size; the detailed description is shown in Section 3.
− As another example, reviewers may want to explore whether adjusting for age and sex of participants influences on the conclusion of a meta‐analysis. Age and sex may act as confounding factors in non‐randomized studies of interventions. Variations in these characteristics across studies can distort the association between an intervention and an outcome of interest. By conducting a sensitivity analysis, the reviewers can compare adjusted ORs (Odds Ratio), which account for age and sex, with unadjusted ORs from primary meta‐analysis.

## 3 How to Undertake a Sensitivity Analysis

1. **Performing a sensitivity analysis based on exclusion**

Most systematic reviewers exclude studies with a high risk of bias, variations in PICO, and outliers in effect size, as described above, when considering a sensitivity analysis.

**FIGURE 1:**
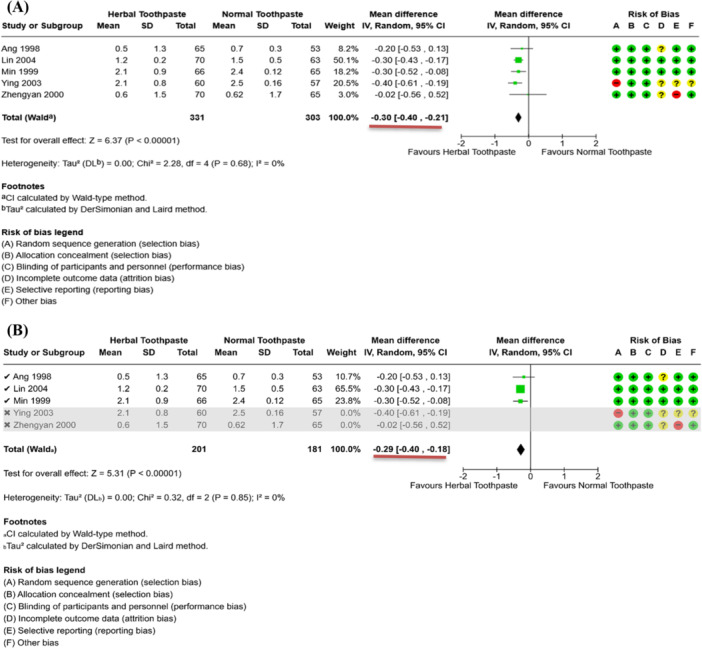
A hypothetical example of a sensitivity analysis omitting two studies with a high risk of bias (A) Primary meta‐analysis (B) Sensitivity analysis (the red bars show the changes in effect size).

The following hypothetical example of a sensitivity analysis which excludes studies is given in Figure 1, showing the comparison of herbal and normal toothpaste. After 2 years of a tooth‐brushing program, the meta‐analysis shows that children who have used herbal toothpaste have a greater reduction in tooth decay than children who have used normal toothpaste (see Figure 1A). The outcome of interest is the mean number of teeth with decay (continuous variable). After excluding two studies with a high risk of bias in a sensitivity analysis, the effect size “MD (Mean Difference) in tooth decay” slightly drops from ‐0.30 [‐0.40, ‐0.21] to ‐0.29 [‐0.40, ‐0.18] as highlighted by red bars in Figure 1B.

The magnitude of the effect size slightly decreases and its confidence interval is narrowed after the sensitivity analysis. As a result, both the primary meta‐analysis and sensitivity analysis suggest that herbal toothpaste is more effective in reducing tooth decay than normal toothpaste. Therefore, the review authors will have more confidence that the findings of their review are reliable and robust even when studies at high risk of bias are included.

**FIGURE 2:**
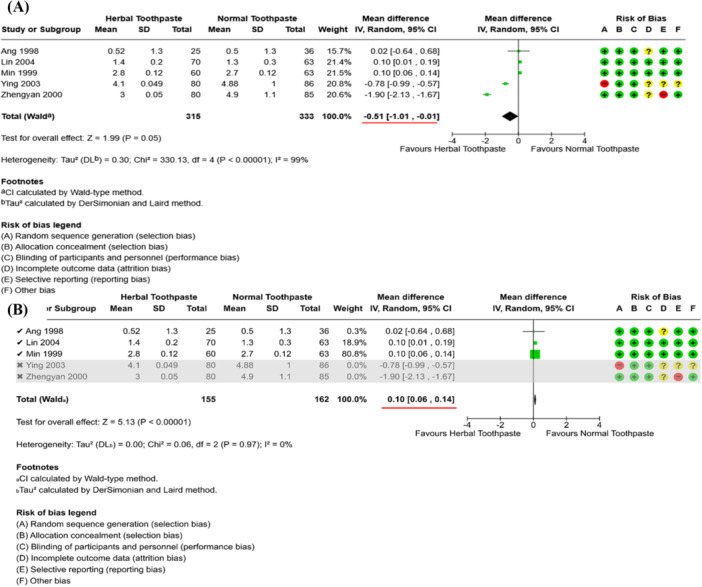
A hypothetical example of a sensitivity analysis of exclusion in which change in both direction and magnitude of the effect size (Red bars) after omitting two studies with high risks of bias (A) Meta‐analysis (B) Sensitivity analysis.

In contrast, the following hypothetical example in Figure 2 shows that children brushing with herbal toothpaste have a significant decrease in tooth decay when compared with children brushing with normal toothpaste (Figure 2A). After excluding two studies with a high risk of bias in a sensitivity analysis, the effect size “MD (Mean Difference)” obviously alters from ‐0.51 [‐1.01, ‐0.01] to 0.10 [0.06, 0.14] as highlighted by the red bars in Figure 2. The results from sensitivity analysis now indicate that the herbal toothpaste is significantly less effective in the reduction of tooth decay than the normal toothpaste.

As a result, the sensitivity analysis demonstrates a major change in both the magnitude and direction of the effect estimate. Therefore, we can conclude that the results of the main meta‐analysis (Figure 2A) are highly influenced by the studies with a high risk of bias. Review authors may decide to conclude that the meta‐analysis's findings have an uncertainty across the varied assumptions and readers should interpret these with caution. Please note that review authors should also take into account the minimally important difference (MID) when deciding whether the sensitivity analysis changes the conclusions. For more information on MID please see Chapter 15 of the Cochrane Handbook [1].

In this example, it should be additionally noted that the two studies with a high risk of bias in Figure 2 are obvious outliers, and review authors may also want to explore whether there are any other reasons, in addition to high risk of bias, for such differing results.

2. **Performing sensitivity analysis when dealing with missing data**

There are many different circumstances where data might be missing. It could be missing statistics such as standard deviations (SD), which may need to be imputed to be able to include the study in the meta‐analysis. Authors may explore the impact of missing data by excluding the study completely from the analysis, or they may want to test the impact of using different SD values. One common example is when authors want to include cluster RCTs in a meta‐analysis, but the trial authors haven't appropriately adjusted for clustering. Review authors can use an intra‐class correlation coefficient (ICC) to recalculate or adjust the sample sizes for clustering. In a sensitivity analysis, authors can investigate how the result might change when different ICC values are used. More information is available in Chapter 23 of the Cochrane handbook [1].

Outcome data can also be missing for participants. Common reasons for missing outcome data are loss to follow‐up and participants missing clinic appointments. As a result, the “missingness” itself is an assumption on which to perform sensitivity analysis.

It is important to classify the types of missingness (see Cochrane Handbook [1] Chapter 10.12.2).

“Missing at random” (MAR): missingness is not related to the actual values of the missing data. For example, a recruited participant fails to attend follow‐up due to a traffic jam.

“Missing not at random” (MNAR): missingness is related to the actual values of the missing data. For example, a recruited person passes away due to the side effects of the drug used in the intervention arm and then is lost to follow‐up.

Before conducting an imputation method, the reviewers should assess whether the missing data is at random or not. When dealing with missing data from primary studies, a previous ‘Methods and Statistics tutorials’ details the different approaches to dealing with missing data and the Intention‐To‐Treat analysis [2]. These approaches are:

i. include data from analyses in which study authors have performed imputations of missing data (if available);
ii. impute missing data yourself; various imputation methods are available (see Cochrane Handbook [1] Chapters 10.12.2 and 10.12.3);
iii. include data from a complete‐case analysis (if available)

A review author may want to use sensitivity analyses to explore whether the approach they used is valid.

3. **Performing sensitivity analysis by exchanging models**

To test the robustness of the conclusion, a sensitivity analysis by exchanging models can be used. This approach allows the authors to investigate the influence of study weights and to assess reliability of the findings across the different statistical models. In addition, it also helps account for between‐study difference mentioned in the above.

**FIGURE 3:**
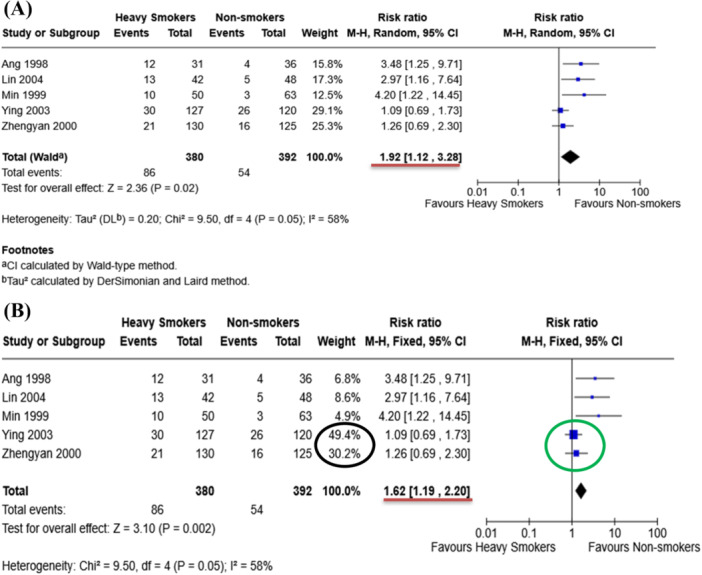
Forest plots showing the change in magnitude of pool estimates (Red bars) after alternating random‐ to fixed‐effect model. (A) Random‐effects model, and (B) Fixed‐effect model in which the green circle indicates the effect size of large trials and the black circle highlights allocating more weight to large trials.

A hypothetical example shows that there is the risk of “Dry Socket” (Alveolar Osteitis), one of the complications after dental extraction, among heavy smokers compared to non‐smokers (Figure 3). By changing from the random‐ to fixed‐effect model, the risk of dry socket in heavy smokers declines from 1.92 [1.12, 3.28] to 1.62 [1.19, 2.20] as shown by the red bars in Figure 3. This means a 30% reduction in the risk of dry sockets in heavy smokers after changing models. This sensitivity analysis demonstrates that the magnitude and precision of the pooled estimate may be implicated with the choice of statistical model.

The differences between random‐effects and fixed‐effect models are explained in detail in Chapter 10 of the Cochrane Handbook [1]. In general, the random‐effects model enumerates heterogeneity of the studies due to differences in sample or research methodology. As a result, the random‐effects model more evenly allocates weights than the fixed‐effect model, allowing relatively more weight to smaller studies. In contrast, fixed‐effect model gives more weight to larger, more precise studies, as highlighted in Figure 3. This assumption is suitable if the studies with similar research methodology involve comparable populations.

However, the decision between these models should be selected based on conceptual considerations rather than a statistical test for heterogeneity, as highlighted in Section 10.10.4.1 of the Cochrane Handbook [1].

## 4 Reporting Sensitivity Analysis

The sensitivity analyses should be reported in reference to Table 1;

**TABLE 1:** Checklist for reporting sensitivity analysis in a systematic review.

Sections of Systematic Review	Explanation
Protocol	− Excluding studies may reduce the amount of data, resulting in less precise estimates. It may also cause results to appear significantly difference, when in fact this has only occurred by chance. Therefore, state only a minimal number of sensitivity analyses. − Sensitivity analysis should be specific to the primary objective of the review [3]
Results	− To avoid confusion with the main meta‐analysis, results of the sensitivity analysis should be presented in a table alongside the main result, rather than in separate forest plots. This allows easier comparison of the findings. − Ensure the results of the sensitivity analysis are described narratively. − Present the results in Summary of Findings (SoF) tables only if findings of sensitivity analysis are notably different from those of the main meta‐analysis. These might be used as to inform the GRADE assessments [1].
Discussion	− Assess the impact of the sensitivity analysis on the robustness of the conclusions, and its implications for research, clinical practice, and policy [1]

## 5 Drawing Conclusions From Sensitivity Analysis

Review authors can compare the findings of the main meta‐analysis with the results of the sensitivity analysis. If the estimate of sensitivity analysis is consistent with the result of the main meta‐analysis, the findings of the review can be considered reliable and robust. Note that minimally important difference (MID) should ideally be considered when making this comparison [4] (see Section 1). If the conclusions based on the MID from the main meta‐analysis aren't changed, then this increases confidence in the findings. Otherwise, when the discrepancy exists, we should state inconsistency among the different assumptions, and evaluate the uncertainty associated with meta‐analysis [1].

## 6 Differences From Subgroup Analysis

Sensitivity analysis is often mistaken for subgroup analysis. The choice of the analyses depends on what the reviewers want to explore. For example, a subgroup analysis can be used to explore the treatment effects on the different age groups. In contrast, a sensitivity analysis is conducted to test the robustness of the overall treatment effect by changing assumptions, such as focusing the treatment effect on the disease high‐risk group out of the study population. Table 2 demonstrates the differences between sensitivity and subgroup analyses [1, 3].

**TABLE 2:** Differences between sensitivity and subgroup analyses.

Aspect	Sensitivity analysis	Subgroup analysis
Timing	− Can be predetermined in the protocol but some are only known post hoc during the review process	− Should be predetermined in the protocol.
Data	− Estimate data based on changing assumptions	− Estimate data from each subgroup
Objective	− Test the robustness of the findings across different assumptions	− Stratify the data based on demographic characteristics, cut‐offs, and thresholds
Comparison	− Informal comparison between sensitivity analysis and primary meta‐analysis	− Formal statistical comparison across the subgroups
Presentation	− Results expressed in tables/text of review, rather than creating separate forest plots.	− Can be demonstrated in the forest plot of main meta‐analysis, or separate forest plots may be created.

## 7 Disadvantages of Sensitivity Analysis

The common pitfalls of the analyses [3] are:

a. A sensitivity analysis is usually conducted based on one assumption at a time while holding other possible statements fixed. However, in real‐life scenarios, the hypotheses are interdependent and concurrently investigated.
b. Only informal comparisons are made between the primary meta‐analysis and the sensitivity analysis, and its interpretation is subjective. Consequently, the validity of the analysis relies on judgment of researchers rather than objective assessment of statistics.
c. Excluding studies may reduce the amount of data, which can make less precise estimate. It may also cause results to appear significantly difference, when in fact this has only occurred by chance. This is why it is important to restrict them and pre‐specify them in the protocol where possible.

## Author Contributions

**Nyan Min Aung:** conceptualization, methodology, writing – original draft, writing – review and editing. **Ivan Jurak:** conceptualization, methodology, writing – original draft, writing – review and editing. **Seemab Mehmood:** writing – review and editing. **Emma Axon:** conceptualization, methodology, supervision, project administration, writing—review and editing.

## Funding

The authors received no specific funding for this work.

## Conflicts of Interest

Emma Axon is employed by Cochrane. Nyan Min Aung is affiliated with the University of Dental Medicine, Mandalay.

## Data Availability

The data that support the findings of this study are available from the corresponding author upon reasonable request.
