# Correction to “Sensitivity Analysis in Meta‐Analysis: A Tutorial”

**DOI:** 10.1002/cesm.70070

**Published:** 2026-01-28

1

N. M. Aung, I. Jurak, S. Mehmood, and E. Axon, “Sensitivity Analysis in Meta‐Analysis: A Tutorial,” *Cochrane Evidence Synthesis and Methods* 4 (2026): 1–7. https://doi.org/10.1002/cesm.70067.

The article category has been corrected from “METHODS ARTICLE” to “TUTORIAL.”

We apologize for this error.

